# Clinico-Epidemiological Profile of Pediatric Diphtheria Cases in a Tertiary Care Hospital in Western India: A Cross-Sectional Study

**DOI:** 10.7759/cureus.91821

**Published:** 2025-09-08

**Authors:** Shivji Ram, Nalini Nawal, Mohan Makwana, Pradeep Kaswan, Souvik Manna

**Affiliations:** 1 Department of Paediatrics, Government Medical College, Barmer, Barmer, IND; 2 Department of Paediatrics, Government Medical College, Pali, Pali, IND; 3 Department of Paediatrics, Dr. Sampurnanand Medical College, Jodhpur, Jodhpur, IND; 4 Department of Community Medicine, Employees State Insurance Corporation (ESIC) Medical College and Hospital, Alwar, Alwar, IND

**Keywords:** clinico-epidemiology, diphtheria, immunization, incidence, myocarditis

## Abstract

Background: Diphtheria is an acute, fatal, bacterial toxin-induced disease and a significant health problem in developing countries. It remains endemic in India, including the state of Rajasthan.

Materials and methods: The present study was a cross-sectional observational study conducted in a tertiary care center of Western India (Jodhpur, Rajasthan) over a period of one year (January to December 2019). All patients in the pediatric age group (up to 18 years of age) who presented with clinical features of suspected diphtheria per the WHO case definition and a positive throat swab for Klebs-Löffler bacillus (KLB) stain were enrolled in the study.

Results: A total of 21,146 patients in the pediatric age group were admitted during the study period, out of which 66 fulfilled the inclusion criteria. The proportion of diphtheria was 3.121 per 1,000 admissions, with the highest number of cases admitted in the winter season from November to February 2019 (42, 63.64%). The male-to-female ratio was 1.87:1, and most cases were from lower socioeconomic strata (43, 65.15%), resided in rural areas (57, 86.36%), were partially immunized (42, 63.64%), and belonged to the 6-14 years age group (49, 74.24%). Most cases presented with pseudomembrane (64, 96.97%), followed by fever (60, 90.91%), dysphagia (57, 86.36%), and neck oedema (51, 77.27%). The most common complications were myocarditis (23, 34.85%), followed by polyneuropathy (17, 25.76%), acute kidney injury (16, 24.24%), bleeding diathesis (15, 22.73%), and multiple organ dysfunction syndrome (MODS) (11, 16.67%). Thrombocytopenia, high levels of blood urea, serum sodium and potassium, and creatine kinase-myocardial band (CK-MB) were significantly associated with poor outcomes, and 23 cases (34.84%) had abnormal ECG findings suggestive of myocarditis. The case fatality rate (CFR) was 18.18% (12/66), ranging from 11.90% among partially immunized cases to 33.33% among unimmunized children.

Conclusion: High immunization coverage for diphtheria among children under five years of age has shifted the age group to 6-14-year-old children who are less immune to the disease. Biochemical parameters such as low platelets, high blood urea, serum sodium and potassium, and raised serum CK-MB are associated with poor outcomes. Myocarditis also carries a poor prognosis.

## Introduction

Diphtheria is an acute, fatal, bacterial toxin-induced disease of ancient times caused by *Corynebacterium diphtheriae*. During 2020, about 1,991 cases of diphtheria were reported in India, with about 28 deaths. Most of the cases reported were from West Bengal (950 cases, one death), Telangana (254 cases and no deaths), Andhra Pradesh (202 cases and no deaths), Rajasthan (148 cases and eight deaths), and Delhi (136 cases and 15 deaths) [1]. *C. diphtheriae* is a Gram-positive, aerobic, non-sporulating, non-capsulated, and nonmotile bacterium around 2µm in length [2]. It is also an exclusive inhabitant of human skin and mucus membranes [3]. There are four known biotypes of *C. diphtheriae*: gravis, mitis, belfanti, and intermedius [4]. The most common type of diphtheria is classic respiratory diphtheria, which can also affect the skin (cutaneous diphtheria). The incubation period of *C. diphtheriae* is two to five days (ranging 1-10 days) [5]. The epidemiology of diphtheria shows that there is an age shift to higher age groups from under-five to 6-14 years, owing to better immunization coverage in under-five children and waning immunity in adults [6-8].

*C. diphtheriae* is not a very invasive organism; the major virulence of *C. diphtheriae* results from the action of its potent exotoxin [9]. The main sign of this infection is a leathery, asymmetrical, greyish-white pseudomembrane over the tonsils, firmly attached to the underlying tissue and bleeding when attempts are made to remove it. The classic and most severe presentation of diphtheria is a respiratory disease with a swollen “bull neck” and a strongly adherent pseudomembrane that obstructs the airways.

A major share of the diphtheria cases occurring in the world each year comes from India. There has been a declining trend in new cases after the introduction of the Universal Immunization Program (UIP) in India, which introduced a pentavalent vaccine (against diphtheria, pertussis, tetanus, hepatitis B, and *Haemophilus influenzae* type B) to be administered at 6, 10, and 14 weeks after birth. Two boosters of diphtheria, pertussis, and tetanus (DPT) are also given at 16-18 months and five years, respectively. Still, around 2,000 to 4,000 cases are reported annually in India (2365, 3380, 5293, 8788, and 9622 cases in 2015, 2016, 2017, 2018, and 2019, respectively) [10]. India alone accounted for 83.3% of the global burden of diphtheria in 2014 and reported 50.17% (3380/6736) of the globally reported cases of diphtheria in 2016. In the last decade, there have been reports of the reemergence of diphtheria from endemic as well as non-endemic states in India as outbreaks, aptly fulfilling the definition of reemerging infections. Most of these outbreaks were characterized by cases in low immunization pockets, especially in Assam (2010), Kerala (2016), and Uttarakhand (2021), among other Indian states [10-12]. Factors such as inadequate vaccination coverage, poor socioeconomic status (SES), overcrowding, poor health-seeking behavior, non-availability, and delay in administration of diphtheria antitoxin further contributed to the re-emergence.

The current study was done in an endemic state with the aim of describing the sociodemographic and clinical profile of all cases of diphtheria reporting to a tertiary hospital during one year. The study also assessed the association of poor outcome (death) with vaccination status at the time of admission.

## Materials and methods

This cross-sectional observational study was conducted in the pediatrics department of a tertiary care hospital in Western India (Jodhpur, Rajasthan). Informed consent from parents/guardians and assent from children less than 18 years was obtained before enrolment in the study. Ethical approval for the study was obtained from the Institute Ethics Committee of Dr. Sampurnanand Medical College, Jodhpur (SNMC/IEC/2020/783-785 dated 13/2/2020), where it was conducted, and the study adhered to the tenets of the Declaration of Helsinki (2024 revision).

The study duration was one year, from January to December 2019. All suspected patients of diphtheria among children and adolescents (age group less than 18 years) who fulfilled the WHO case definition for diphtheria were screened for the study (presenting with pharyngitis; nasopharyngitis; tonsillitis; laryngitis; or adherent pseudomembrane of the pharynx, tonsil, larynx or nose and other clinical features consistent with diphtheria like fever, throat pain, difficulty in swallowing, stridor, voice change, nasal regurgitation and bull neck) [13]. In addition, mild cases without a pseudomembrane and those with nonhealing ulcers with a travel history to countries with endemicity for the disease or countries with diphtheria outbreaks were also screened. The screening was done using a throat swab with Albert's stain, and only those with a positive throat swab for Klebs-Löffler bacillus (KLB) were included in the study. Although culture remains the gold standard for diagnosis, Albert's stain is much faster for clinical decision making, hence it was taken as an inclusion criterion for the current study. Those who were negative for KLB staining were not included in the study. It is usual practice to initiate treatment based on throat swab positivity without waiting for culture confirmation, as any delay in management could be fatal. Further, Elek’s test (immunodiffusion test) can be used for toxin confirmation, but was not done due to the unavailability of reference laboratory facilities in the study area.

A predesigned proforma was used to record information about sociodemographic factors, a detailed medical history, and a general physical and systemic examination (see Appendix A). All patients were subjected to basic laboratory investigations on the day of admission, and results were recorded in the proforma after obtaining the results from the laboratory. All relevant investigations, such as complete blood count (CBC), erythrocyte sedimentation rate (ESR), renal function tests (RFTs), and urine for albumin and sugar, were performed on admission. Electrocardiography (ECG) was done on the first day and daily thereafter to monitor for potential cardiac complications, specifically detecting abnormalities in heart rhythm and electrical conduction, as diphtheria can lead to myocarditis [14]. Two-dimensional echocardiography (2D-ECHO) and creatine kinase-myocardial band (CK-MB) were done on the 10th day of onset of illness, as indicated clinically or after abnormal ECG findings. Troponin-I and T, although more sensitive and specific than CK-MB, were not done, as the study participants belonged to a lower SES, and the cheaper tests available under government subsidy were only advised (e.g., CK-MB).

Immunization status was documented as per the information given by the parents or legal guardian and confirmed by the immunization (Mother Child Tracking System, or MCTS) card. Those who had received three primary doses at four- to six-week intervals starting at one and a half months of age, followed by booster doses at 18 months and five years, were recorded as fully immunized. Those who had not received even a single dose were considered unimmunized. Patients who did not receive a full course of vaccination appropriate for their age (all three primary doses and two booster doses) were considered partially immunized. These case definitions were formulated for the purpose of the study, keeping in view the good coverage of DPT up to five years of age. The tetanus-diphtheria (Td) booster dose is given for 10- and 16-year-old adolescents as well; however, adolescent vaccination coverage is poor in most states in India, including Rajasthan. Hence, adolescent doses were not included in the definition of fully immunized.

Those patients who had been in close contact with a clinical case of diphtheria per the WHO case definition and confirmed microbiologically were considered as having a history of contact with a diphtheria case.

Patients were monitored for all possible complications, and treatment was done according to standard guidelines. Management was given in terms of initial supportive care, including bed rest, airway management, isolation, and a specific antitoxin. Patients were discharged on clinical improvement, completion of antibiotic therapy, and negative repeat KLB on the 14th day of therapy.

Statistical analysis was performed by the IBM SPSS Statistics for Windows, Version 26 (Released 2020; IBM Corp., Armonk, New York, United States). In the first step, a descriptive analysis was carried out to characterize the sociodemographic features of the study participants as percentages and proportions. In the second step, appropriate tests were applied, including a chi-square test for categorical variables and a t-test for continuous variables to calculate test statistics and p-values. The level of significance was kept at <0.05 for finding an association between variables.

## Results

A total of 21,146 patients in the pediatric age group were admitted during a study period of one year. Out of these, 521 cases that presented with clinical features of suspected diphtheria per the WHO case definition were enumerated for the study and screened by throat swab for KLB. A total of 66 (12.67%) participants had positive throat swabs by Albert's stain and were included in the study. Culture was used for confirmation of diagnosis, but was not an inclusion criterion for the study, as culture results came after one to two days, and treatment was started immediately for most cases based on throat swab results only. Any delay in starting the treatment or waiting for culture results could be fatal for the patient. The rationale of excluding the 455 patients who satisfied the WHO case definition but failed the throat swab is that causes other than diphtheria (streptococcal pharyngitis, Epstein-Barr virus (EBV), *Candida*, etc.) can also present with throat membranes. The WHO case definition has poor specificity, and combining it with a throat swab increases the specificity further.

Cases of diphtheria were admitted throughout the year, but the maximum number of patients (20, 30.30%) was reported in the month of November 2019, and the minimum in April (1, 1.52%). The overall incidence rate was 3.121 per 1000 admissions per year (Table 1).

**Table 1:** Incidence rate of diphtheria in admitted cases during the study period (January-December 2019) IPD: in-patient department

Months (2019)	Total cases enrolled (IPD) (N=21146)	Cases of diphtheria (N=66)	Incidence rate (per 1000 admissions)
January	1775 (8.39%)	07 (10.61%)	03.944
February	1804 (8.53%)	06 (9.09%)	03.326
March	1603 (7.58%)	05 (7.58%)	03.119
April	1731 (8.19%)	01 (1.52%)	00.578
May	1450 (6.86%)	02 (3.03%)	01.379
June	1429 (6.76%)	0	0
July	1619 (7.66%)	04 (6.06%)	02.471
August	1667 (7.88%)	02 (3.03%)	01.200
September	2039 (9.64%)	04 (6.06%)	01.962
October	2334 (11.04%)	06 (9.09%)	02.571
November	1620 (7.66%)	20 (30.30%)	12.346
December	2075 (9.81%)	09 (13.64%)	04.337
Total	21146 (100%)	66 (100%)	03.121

Out of the total of 66 diphtheria patients, 43 (65.15%) cases were male, whereas 23 (34.85%) were female, resulting in a male-to-female ratio of 1.87:1. Most of the cases (38, 57.58%) were in the age group of 6-10 years with similar distributions among male (24, 55.81%) and female children (14, 60.87%). The mean age of total enrolled cases was 8.17±3.35 (2-17) years, whereas for males it was 7.65±3.35 (2-15) and for females it was 9.13±3.21 (4-17) years. The difference was not statistically significant (t=-1.73, p=0.087) (Table 2).

**Table 2:** Demographic profile of diphtheria cases (N=66) p: probability; t: t-statistics; χ^2^: chi-square statistics

Age groups (years)	Male (N=43), n (%)	Female (N=23), n (%)	Total (N=66), n (%)	Test statistics, p-value
0-5	11 (25.58)	2 (8.69)	13 (19.70)	χ^2^=4.501, p=0.212
6-10	24 (55.81)	14 (60.87)	38 (57.58)	
11-15	8 (18.60)	6 (26.09)	14 (21.21)	
>15	0	1 (4.35)	01 (1.52)	
Mean age	7.65±3.35	9.13±3.21	8.17±3.35	t=-1.73, p=0.087
Religion				
Hindu	38 (88.37)	18 (78.26)	56 (84.85)	χ^2^=2.415, p=0.299
Muslim	4 (9.30)	5 (21.74)	09 (13.64)	
Sikh	1 (2.33)	0	01 (1.52)	
Residence				
Rural	38 (88.37)	19 (82.61)	57 (86.36)	χ^2^=0.075, p=0.784
Urban	5 (11.63)	4 (17.39)	09 (13.64)	
Socioeconomic status				
I (upper)	2 (4.65)	0	02 (3.03)	χ^2^=4.104, p=0.392
II (upper middle)	3 (6.97)	1 (4.35)	04 (6.06)	
III (lower middle)	1 (2.33)	1 (4.35)	02 (3.03)	
IV (upper lower)	7 (16.28)	8 (34.78)	15 (22.73)	
V (lower)	30 (69.77)	13 (56.52)	43 (65.15)	
Immunization status				
Complete	3 (6.97)	0	03 (4.55)	χ^2^=1.716, p=0.424
Partially	27 (62.79)	15 (65.22)	42 (63.64)	
Unimmunized	13 (30.23)	8 (34.78)	21 (31.82)	

Most of the patients were from the Hindu community (56, 84.85%) and resided in rural areas (57, 86.36%), with a rural-to-urban ratio of 6.3:1. Most children had lower SES (43, 65.15%) followed by upper-lower (15, 22.73%) and upper-middle (4, 6.06%) according to the modified Kuppuswamy socioeconomic scale [15]. As far as contact history is concerned, 64 cases (96.97%) had no history of contact with any confirmed case of diphtheria, compared to only two (3.03%) cases with a positive history of contact. In the current study, 12 patients (18.18%) expired, and 54 patients (81.82%) were discharged after recovery. Out of the 12 who expired, nine were males and three were females; two were under two years of age, eight were between two and six years old, and the remaining two were over six years old.

In the survival group, all but one (53, 98.15%) of the diphtheria patients had a hospital stay of 11-14 days with a mean duration of 14.98 (±7.21) days. Among the expired patients, 10 patients (83.3%) died in the first five days, followed by two (16.67%) in 6-14 days, with a mean duration of stay of 3.28 (±3.91) days in these patients. The mean duration of stay was much lower among the expired patients, and the difference was statistically significant (Table 3).

**Table 3:** Comparison of characteristics among recovered and expired patients (N=66) * statistically significant; WBC: white blood count; ESR: erythrocyte sedimentation rate; CK-MB: creatine kinase-myocardial band

Variables	Recovered (N=54)	Expired (N=12)	Total (N=66)	Range	t-stat, p-value
Duration of stay (days)	14.98 (± 7.21)	3.28 (± 3.91)	12.85 (± 8.11)	0.33-67	5.425, 0.000*
Age (years)	8.69 (± 3.21)	5.83 (± 3.04)	8.17 (± 3.35)	2-17	2.810, 0.007*
Hemoglobin (g/dL)	11.51 (±1.40)	11.22 (±1.22)	11.46 (±1.36)	8.10-13.70	0.685, 0.496
WBC (per mm^3^)	14809.81 (±6198.19)	18050.0 (±6745.06)	15399 (±6373)	6340-37310	-1.613, 0.112
Neutrophils (%)	71.30 (±11.41)	71.42 (±11.18)	71.31 (±11.28)	42.00-97.00	-0.033, 0.974
Lymphocytes (%)	21.91 (±10.53)	24.75 (±10.75)	22.42±10.54	3.00-48.00	-0.843, 0.403
Platelet counts (per cu mm)	2,16,407.41 (±1,11,655.39)	1,16,000 (±1,15,774.08)	198152 (±118138)	22000-539000	2.800, 0.007*
ESR (mm/first hour)	26.31 (±10.34)	27.08 (±10.97)	26.45 (±10.37)	8.00-54.00	-0.230, 0.818
Urea (mg/dL)	36.65 (±28.39)	56.92 (±26.23)	40.33±28.90	14.00-147.0	0.483, 0.027*
Creatinine (mg/dL)	1.01 (±0.73)	1.14 (±0.48)	1.03±0.68	0.36-5.56	0.918, 0.581
CK-MB (IU/L)	64.80 (±77.62)	146.17 (±157.88)	79.59±100.65	18-612	-2.648, 0.010*
Sodium (mmol/L)	143.74 (±4.78)	147.25 (±7.79)	144.37±5.54	133.0-166.0	-2.031, 0.046*
Potassium (mmol/L)	4.43 (±0.53)	4.88 (±0.63)	4.51±0.56	3.30-5.90	-2.569, 0.013*

In addition, the expired patients also had significantly lower ages and lower platelet counts, along with higher blood urea, CK-MB, and serum sodium and potassium levels (Table 3). The mean CK-MB level among 66 cases was 79.59 (±100.65) IU/L. The mean CK-MB level in patients with myocarditis (suggested by ECG findings) was 143.61 (±149.96) IU/L compared to 45.34 (±21.32) IU/L in those who had normal ECGs, and the difference was statistically significant (t-stat:-3.13, p=0.005).

**Figure 1:**
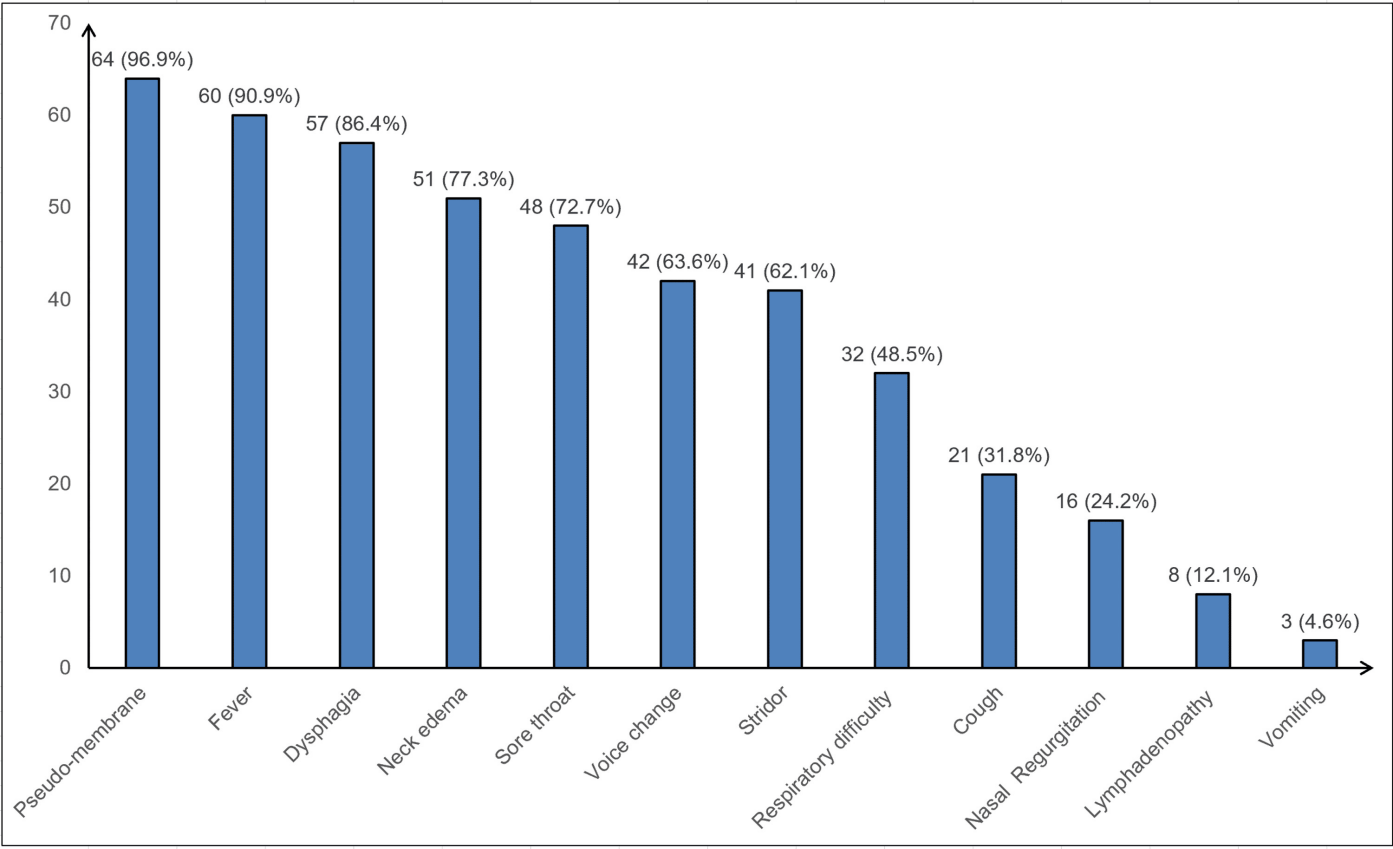
Distribution of cases according to clinical presentation on admission (N=66)

The clinical profile revealed that the most common presentation was pseudo-membrane: tonsillo-pharyngeal (60, 90.91%), pharyngo-laryngeal (3, 4.55%), and nasal membranes (1, 1.55%). Other common clinical features were fever (60, 90.9%), dysphagia (57, 86.36%), neck edema (51, 77.27%), and sore throat (48, 72.73%) (Figure 1).

Similarly, the most common complications detected were myocarditis (23, 34.85%), polyneuropathy (17, 27.42%), acute kidney injury (16, 24.24%), bleeding (15, 22.73%), and multiple organ dysfunction syndrome (MODS; 11, 16.67%).

The ECG findings showed that 43 of the diphtheria cases (79.63%) had normal ECGs during the period of hospitalization and were discharged, whereas 23 (34.85%) cases had various ECG abnormalities, suggestive of myocarditis (Table 4).

**Table 4:** ECG findings and immunization status in cases of diphtheria segregated by outcome (N=66) ECG: electrocardiography; AV: atrioventricular

ECG findings	Discharged (N=54), n (%)	Expired (N=12), n (%)	Total (N=66), n (%)
Normal ECG	43 (79.63)	0 (0.00)	43 (65.15)
Total abnormal ECG finding	11	12	23 (34.84)
Sinus tachycardia	6 (11.11)	4 (33.3)	10 (15.15)
Prolonged PR intervals	2 (3.7)	2 (16.67)	4 (6.06)
ST-segment depression	0	1 (8.33)	1 (1.52)
T wave inversion	1 (1.85)	2 (16.67)	3 (4.55)
Bundle branch block	2 (3.70)	2 (16.67)	4 (6.06)
AV block	0	1 (8.33)	1 (1.52)
Completely immunized	03 (5.55)	0	03 (4.55)
Partially immunized	37 (68.51)	5 (41.67)	42 (63.64)
Unimmunized	14 (25.93)	7 (58.33)	21 (31.82)

Abnormal ECG findings included sinus tachycardia (10, 15.15%), prolonged PR intervals (4, 6.06%), ST segment depression (1, 1.52%), T wave inversion (3, 4.55%), bundle branch block (4, 6.06%), and atrioventricular (AV) block (1, 1.52%; Table 4). Out of the 12 patients who expired, the maximum had sinus tachycardia (4, 33.3%), followed by prolonged PR interval, bundle branch block, and T wave inversion (2 each, 16.67%). Most of the children with diphtheria were partially immunized (42, 63.64%), but 21 were unimmunized (31.82%). Only three (4.55%) patients received complete age-appropriate immunization, out of which two were 15 years old and one was five years old. However, out of 42 (63.64%) partially immunized patients, 33 (78.57%) were in the age group of 6-14 years, followed by eight (19.05%) in the 0-5 years group, and only one in the 14-18 years group. A total of 21 (31.82%) patients were not immunized for diphtheria, out of which 16 (76.19%) were 6-14 years old, whereas four (19.05%) were under five, and the remaining one was 15 years old.

Based on gender, the proportion of male and female patients who were partially immunized (62.80% vs. 65.22%) and the proportion who were not immunized (30.23% vs. 34.78%) were similar, and the difference was not statistically significant. Completely immunized cases belonged to socioeconomic classes I and II only, whereas unimmunized cases belonged to classes IV and V.

Favorable outcomes were documented in completely immunized children, all of whom recovered fully and were discharged. In the partially immunized group, 37 patients (88.10%) recovered, whereas the remaining five (11.90%) succumbed to death. The least favorable outcome was observed among unimmunized cases, for whom a case fatality rate of 33.33% (7/21) was observed. The difference in the outcomes in the three groups was statistically significant (p-value <0.05).

In the study cases, the most common complication observed was myocarditis (23, 34.85%), followed by polyneuropathy (17, 25.76%), acute kidney injury (16, 24.24%), bleeding diathesis (15, 22.73%), and MODS (11, 16.67%) during the period of hospitalization. Complications were proportionally higher among the unimmunized compared to the partially immunized and completely immunized cases. A statistically significant difference was observed in polyneuropathy (47.62% vs. 16.67%, p=0.017) and acute kidney injury (42.86% vs. 16.67%; p=0.044) in the unimmunized group compared to the partially immunized group (Table 5).

**Table 5:** Distribution of diphtheria cases according to complications and immunization status * statistically significant; p-value: probability; χ^2^: chi-square statistics; MODS: multiple organ dysfunction syndrome

Complications	Completely immunized (N=3), n (%)	Partially immunized (N=42), n (%)	Unimmunized (N=21), n (%)	Total (N=66), n (%)	χ^2^, p-value
Myocarditis	01 (33.33)	13 (30.95)	09 (42.86)	23 (34.85)	0.87, 0.65
Polyneuropathy	0	07 (16.67)	10 (47.62)	17 (25.76)	8.10, 0.017*
Kidney injury	0	07 (16.67)	09 (42.86)	16 (24.24)	6.24, 0.044*
Bleeding	01 (33.33)	08 (19.05)	06 (28.57)	15 (22.73)	0.92, 0.629
MODS	0	05 (11.90)	06 (28.57)	11 (16.67)	3.43, 0.180

## Discussion

The current study showed that three-fourths of the cases belonged to the 6-14 years age group, in consonance with previous studies from India showing an increasing age of onset [16]. Two-thirds of the cases were admitted in the winter season (November to February), followed by the rainy season (July to October) and summer (March to June), with a seasonal incidence of 5.988, 2.051, and 1.269, respectively, per 1,000 admitted patients. A study from Rajkot, Gujarat, reported a decreasing trend in the diphtheria case rate over time, which was above 6 per 1,000 admissions before 1989 and decreased to less than 1 per 1,000 admissions during 1993-1997 [17]. However, the rate increased from 2.8 in 1998 to 4.8 per 1,000 admissions in the year 2001. The higher incidence of diphtheria in winter in the current study might be due to environmental factors leading to greater incidence, better health-seeking behavior, and higher reporting of cases. Another study from Rajasthan, India, also reported this seasonal fluctuation, with the maximum number of cases occurring in the fourth quarter from October to December [18]. The reasons cited for this seasonal increase during winter or spring were overcrowding and poor personal hygiene [19]. Previous studies had also reported higher numbers of diphtheria cases and other acute respiratory infections during the rainy season [20,21]. The slight seasonal variation and peak incidence of disease in different studies might be due to regional geographical and climatic conditions.

In the current study, the male-to-female ratio was 1.87:1, which is in concordance with previous studies reporting a male preponderance [11,22]. However, studies from South India have also reported a higher incidence among females [23]. Thus, the gender predilection is actually dependent on social factors and is not due to biological differences between the sexes. The association of diphtheria with rural areas and poor SES reported in the current study is also intuitive, given that the modifiable risk factors of the disease are poor vaccination coverage and lack of knowledge [24].

Out of the 66 patients in the study, 12 patients expired, and 54 were discharged after recovery and completion of treatment. The mean duration of their hospital stays was significantly lower among the patients who had poor outcomes (death). Similar to our study, previous studies had reported that the median length of stay in an acute care facility was eight days for hospitalizations with unspecified diphtheria and 5.5 days for complicated diphtheria [25].

In the current study, two-thirds of the diphtheria cases were partially immunized, and the remaining cases were unimmunized, with the maximum number being from lower SES. The coverage for the first, second, and third doses of the DPT vaccine was 68.20%, 39.40%, and 21.20%, respectively. The overall case fatality rate was 18.18%, ranging from 11.90% among partially immunized cases to 33.33% among unimmunized cases. Previous studies had reported a CFR ranging from 5% to 10%, reaching up to 30% among unimmunized children [26,27]. Some of the prognostic factors leading to poor outcomes in the current study were thrombocytopenia, high levels of blood urea, and elevated serum sodium and potassium, as reported by previous studies [28,29]. The enzyme CK-MB was also significantly higher among those with ECG findings suggestive of myocarditis. The ECG findings reported in previous studies were sinus tachycardia (68.3%), T wave inversion (20%), ST segment depression (13.3%), and right bundle branch block (5%), similar to the findings of the current study [14].

Also, mortality and complications were proportionally higher among the unimmunized compared to the partially immunized and immunized cases, with common complications including myocarditis, polyneuropathy (palatal palsy), acute kidney injury, bleeding, and MODS. Although the study did not collect data on duration of illness before hospitalization, it is prudent to assume that the high rate of complications/mortality in some groups might be due to late presentation to hospital/medical facilities or other social factors like poor health-seeking behaviour. The cause of death in all 12 patients was myocarditis with airway obstruction.

The study had a few limitations. A hospital-based study of a small sample of cases from a tertiary care center might not constitute an actual reflection of community incidence. A small sample size, potential selection bias, and limited external validity are important limitations. Also, the study excluded negative reported cases of KLB smear, and a culture was not performed to rule out diphtheria. Some cases of smear-negative diphtheria might have been missed, which could have been detected by stringent clinical case definitions or culture confirmation. The study missed a chance to find the strongest predictors of death because it did not use multivariate analysis. Further studies should include multivariate analysis to give clearer guidance on predictors of death and severity of illness.

## Conclusions

The incidence of diphtheria reported in the study was 3.121 per 1,000 admissions per year. There was a seasonal trend, with the maximum number of cases in the winter and monsoon months. The most common presenting features were pseudomembrane, followed by fever, dysphagia, and neck oedema. The most common complications were myocarditis, followed by polyneuropathy (palatal palsy), kidney injury, bleeding, and MODS. The case fatality rate was 18%, ranging from 11% to 33% in partially immunized and unimmunized children. Low platelet counts, high blood urea, and elevated serum sodium and potassium were associated with poor outcomes. In diphtheria with myocarditis, CK-MB levels and ECG abnormalities had good prognostic value.

## Appendices

Appendix A

Study Questionnaire

**Table 6:** Study proforma for clinico-epidemiological profile of paediatric diphtheria in a tertiary care hospital of Western Rajasthan M: male; F: female; LAMA: left against medical advice; DAMA: discharged against medical advice; DPT: diphtheria pertussis tetanus; B1: first booster; B2: second booster; H/O: history of; MODS: multiple organ dysfunction syndrome; KLB: Klebs-Löffler bacillus; CBC: complete blood count; HB: haemoglobin; TLC: total leucocyte count; PLT: platelet; ESR: erythrocyte sedimentation rate; Na+: sodium; K+: potassium; CK-MB: creatine kinase-myocardial band; ECG: electrocardiography; 2D-Echo: two-dimensional echocardiography; HR: heart rate; RR: respiratory rate; BP: blood pressure; SpO_2_: partial pressure of oxygen

Patient Name-………………………	Date of birth …………….
Age-……. (In years)	Gender- M/F…………
Address: ………………………………	Registration Number: ……………..
Religion: ……………………	Mobile No +91-……………………..
Date of Admission: ………	
Socioeconomic Status……………..	Education of head of family…………...........
	Occupation of head of family………………
	Monthly income of family……………….....
Duration of hospital stay………. (In days)	Duration of Illness…………
Outcome:	(DISCHARGE/LAMA/DAMA/ABSCOND/EXPIRED/REFERRED)
Immunization status	[complete/incomplete/partially immunized]
DPT1…	DPT2….
DPT3…….	DPT- B1…… DPT- B2……
H/O Contact…….	
History:	
Clinical Features	Details
Fever	
Sore throat	
Membrane (site)	
Neck edema/Bull neck	
Stridor	
Throat pain/dysphagia	
Voice change	
Nasal regurgitation	
Others	
Complications:	
Complications	Details
Myocarditis	
Polyneuropathy	
Kidney injury	
Bleeding	
MODS	
Laboratory Investigations	
Investigations	Day-1 (admission) Day-14 (or As and when required)
Throat swab stain (KLB day 1^st^ and day 14^th^)	
CBC (Hb, TLC, PLT)	
ESR	
Urea	
Creatinine	
Na+	
K+	
CKMB	
ECG (Daily)	
2D-ECHO (if ECG finding/Clinical/Day 10 from onset of illness)	
Proteinuria	
Treatment given…………………….	
Vitals monitoring (Every 12 hours):-	
Temp	
HR	
RR	
BP	
SpO2	
